# Supplementary material for: Children from the poor families seem to grow up earlier: An examination of how family economy stress links to career exploration
Source: PLoS One. 2024 Oct 29;19(10):e0310866. doi: 10.1371/journal.pone.0310866 (PMC11521272; doi:10.1371/journal.pone.0310866)
Supplement: Attachment — Submitted filename: review comments.docx [file pone.0310866.s002.docx]

**Abstrac**t

The opening sentence in the abstract has no connection and has to be improved.

**Introduction**

the gap in using love for money is confusing and not properly addressed. The authors should justify why they want to use money worship as a mediator based on either theory or previous studies.

**Hypotheses development**

What is the direction of the hypotheses you have set especially the intervening variables?

**Methods**

Justify why a 66.67% response rate can be used to generalize when you retrieved lower than your minimum sample size. Which criteria did you use to select participants for the survey?

Consider the choice of a bipolar scale as it is difficult to add positive to negative. That is adding SD to SA is not scientific. I suggest authors read on unipolar scales.

Why did the researchers introduce control in the methods without arguing for them in the introduction or conceptual framework?

**Discussion**

The researchers mentioned discussions but failed to present anything under them.

More concrete examples of how the findings could be implemented in practice would be beneficial.

The section could include more discussion on the practical significance of the findings, not just statistical significance.
