# Supplementary material for: Children from the poor families seem to grow up earlier: An examination of how family economy stress links to career exploration
Source: PLoS One. 2024 Oct 29;19(10):e0310866. doi: 10.1371/journal.pone.0310866 (PMC11521272; doi:10.1371/journal.pone.0310866)
Supplement: Attachment — Submitted filename: Response to Reviewers.docx [file pone.0310866.s003.docx]

**Response to Reviewers**

Thank you for your valuable feedback. We have modified the manuscript according to your opinions and answered the questions raised by reviewers point by point all with the blue context like here. It is appreciated to be recognized by reviewers.

**Reviewer #1:**

Thank you for the opportunity to review the paper entitled, “Children from the Poor seems Grow up Earlier? The Mediating Role of Love for Money in Linking Family Economy Stress to Career Exploration: A Moderated Mediation Model.” I recommend you to carefully review again and revise format throughout the manuscript to follow APA 7th Style. Though I did not list all the format errors, please carefully consider these for your revisions (e.g., past tense).

A: Thank you for your thorough review and valuable feedback on our manuscript. I have carefully reviewed and revised the manuscript to ensure it adheres to the APA 7th Style format throughout. This includes addressing any formatting errors and ensuring consistency in style and tense (e.g., using past tense where appropriate). I have meticulously gone through each section to correct any issues and align the manuscript with the required standards.

Thank you once again for your valuable input and guidance. I have made the necessary revisions and am resubmitting the manuscript for your further review.

**Reviewer #2:**
After reading your manuscript, a series of minor issues need to be addressed.
The text is well written and the results are adequately presented and referenced. I recommend the current manuscript for publication, after minor revisions.

A: Thank you for your thorough review and constructive feedback on my manuscript. I appreciate your positive comments and the recommendations provided to enhance the clarity and quality of the paper. Here is my response to the suggested revisions:

Q1. Please re-adjust your title in accordance to your results

A: We have carefully considered your suggestion regarding the title of the paper and have adjusted it to better reflect the results and key findings of our study. The revised title is: " Children from the Poor Families Seem to Grow Up Earlier: An Examination of How Family Economy Stress Links to Career Exploration".

We believe this new title more accurately encapsulates the primary focus and findings of our research. Thank you once again for your insightful comments. We look forward to any further feedback you may have.

Q2. Please make a clearer depiction of Introduction vs. your literature review

A: Thank you for your constructive feedback and the suggestion to make a clearer distinction between the Introduction and the Literature Review sections.

We have revised the manuscript to distinctly separate these sections. The Introduction now focuses on the background, significance, and objectives of our study, providing a clear overview of the research question and its importance. The Literature Review has been expanded to include a more comprehensive discussion of relevant theories and prior research, establishing the context for our hypotheses and highlighting gaps that our study aims to address.

Q3. Please add some literature cited thresholds when discussing your results

A: We have revised our manuscript to include relevant literature-cited thresholds to better support our discussion and enhance the robustness of our results. This involved referencing key studies and established benchmarks that align with our findings, providing a clearer framework for interpretation.

Reference:

Bauer, D. J., Preacher, K. J., & Gil, K. M. (2006). Conceptualizing and testing random indirect effects and moderated mediation in multilevel models: new procedures and recommendations. *Psychological Methods*, *11*(2), 142-163.

Dawson, J. F., & Richter, A. W. (2006). Probing three-way interactions in moderated multiple regression: development and application of a slope difference test. *Journal of Applied Psychology*, *91*(4), 917-926.

Dawson, J. F. (2014). Moderation in management research: What, why, when, and how. *Journal of Business and Psychology*, *29*(1), 1-19.

Q4. As a suggestion, maybe add a table format where you explain the status of your hypotheses, for a clearer view

A: Thank you for your insightful suggestion regarding the inclusion of a table to explain the status of our hypotheses. We appreciate your recommendation and acknowledge the value it could add to the clarity of our manuscript. However, given that our article already includes eight tables, we were mindful of the overall length and complexity. We have ensured that all our hypotheses are thoroughly supported by the data, and this is detailed within the text. Nonetheless, we will certainly consider this format in future papers or supplementary materials to enhance clarity and accessibility of our findings.

Thank you once again for your valuable feedback.

**Reviewer #3:**

Abstract
The opening sentence in the abstract has no connection and has to be improved.

A: Thank you for your insightful feedback. We appreciate your observation regarding the opening sentence of the abstract and understand the need to improve its connection to the rest of the content. We have revised the opening sentence to better align with the study's focus and provide a smoother transition into the main objectives and findings of the research.

Introduction
the gap in using love for money is confusing and not properly addressed. The authors should justify why they want to use money worship as a mediator based on either theory or previous studies.

A: Thank you for your detailed review and constructive feedback. We understand the importance of justifying this choice based on theoretical grounds or previous research.

The concept of "love of money" as a mediator is grounded in Social Cognitive Career Theory (SCCT) and supported by several studies that highlight the role of financial attitudes in shaping career behavior. Specifically, SCCT posits that individual career development is influenced by personal cognitions and environmental factors, including economic conditions and financial attitudes (Lent & Brown, 1996).

Research has shown that financial attitudes, such as the love of money, significantly impact career-related decisions and behaviors (e.g., Hu et al., 2022; Matricano & Sorrentino, 2019). For instance, Tang et al. (2006) demonstrated that love of money influences job choice, motivation, and work behavior, indicating its strong motivational effect on career exploration. Similarly, other studies have found that economic pressures can enhance the value placed on money, which in turn drives individuals to seek financially rewarding careers (Elder et al., 2020; Tang et al., 2005).

In our study, we propose that family economic stress increases the emphasis on financial security and success, thereby fostering a love of money among vocational students. This heightened financial motivation, in turn, drives proactive career exploration as students seek to alleviate financial pressures and achieve economic stability. By examining love of money as a mediator, we aim to bridge the gap between economic hardships and career exploration, providing a nuanced understanding of how financial attitudes influence vocational behaviors.

Thank you once again for your valuable input. We look forward to your further comments and suggestions.

Reference:

Elder, G. H., Robertson, E. B., & Ardelt, M. (2020). Families under economic pressure. In *Families in troubled times* (pp. 79-103). Routledge.

Hu, S., Hood, M., Creed, P. A., & Shen, X. (2022). The relationship between family socioeconomic status and career outcomes: A life history perspective. *Journal of Career Development*, *49*(3), 600-615.

Lent, R. W., & Brown, S. D. (1996). Social cognitive approach to career development: An overview. *The Career Development Quarterly*, *44*(4), 310-321.

Matricano, D., & Sorrentino, M. (2019). Does love money affect vocational behavior? Empirical evidence from Italian entrepreneurs. *International Journal of Business and Management*, *14*(7), 110-123.

Tang, T. L. P., Sutarso, T., Akande, A., Allen, M. W., Alzubaidi, A. S., Ansari, M. A., & Vlerick, P. (2006). The love of money and pay level satisfaction: Measurement and functional equivalence in 29 geopolitical entities around the world. *Management and Organization Review*, *2*(3), 423-452.

Tang, T. L. P., Tang, D. S. H., & Luna‐Arocas, R. (2005). Money profiles: The love of money, attitudes, and needs. *Personnel Review*, *34*(5), 603-618.

Hypotheses development
What is the direction of the hypotheses you have set especially the intervening variables?

A: Thank you for your insightful feedback. We have revised our hypotheses to highlight the direction of the hypotheses. (It's highlighted in blue in the manuscript)

Methods
1. Justify why a 66.67% response rate can be used to generalize when you retrieved lower than your minimum sample size. Which criteria did you use to select participants for the survey?
2. Consider the choice of a bipolar scale as it is difficult to add positive to negative. That is adding SD to SA is not scientific. I suggest authors read on unipolar scales.
3. Why did the researchers introduce control in the methods without arguing for them in the introduction or conceptual framework?

A1: Thank you for your constructive feedback and for raising the important point regarding the response rate and participant selection criteria.

The response rate of 66.67%, while seemingly moderate, is actually within an acceptable range for social science research, particularly in survey studies involving multiple time points. Research has shown that response rates between 50% and 70% are generally considered satisfactory for ensuring the validity and reliability of survey results (Baruch & Holtom, 2008). In addition, our study uses a three-stage lag design, which is conducive to better observe the causal relationship and dynamic changes among variables. However, we are also aware that this design inevitably increases the turnover rate of questionnaires. After the investigation, our research team conducted strict data cleaning and screening, including checking for missing and incorrect responses. The data were then matched across the three stages of the questionnaire, resulting in the final effective sample size. We believe that these measures adequately address the concern regarding the response rate and selection criteria, supporting the validity and generalizability of our findings

Reference:

Baruch, Y., & Holtom, B. C. (2008). Survey response rate levels and trends in organizational research. *Human relations*, *61*(8), 1139-1160.

A2: Our decision to use a bipolar scale was based on its widespread acceptance and extensive use in similar research studies (Lamm et al. 2020; Spector, 1992;). Bipolar scales, which measure attitudes and perceptions along a continuum from strongly disagree (SD) to strongly agree (SA), are commonly employed in social science research to capture the full spectrum of respondent attitudes and opinions (Cabooter et al., 2016; Gure, 2015). This approach has been validated in numerous studies and provides valuable insights into the relative strengths of participants' responses.

We acknowledge that the use of bipolar scales has certain limitations, such as the potential complexities in interpreting combined positive and negative responses. However, this method remains a robust tool for assessing nuanced attitudes and has been effectively utilized in a variety of empirical studies (e.g., Cheung & Jin, 2016; Lemrová et al., 2014; Lu & Jia, 2022; Wadsworth & Compas,2002).

We appreciate your suggestion to explore unipolar scales and will certainly consider this approach in future research to compare its effectiveness against the bipolar scale method.

Reference:

Cabooter, E., Weijters, B., Geuens, M., & Vermeir, I. (2016). Scale format effects on response option interpretation and use. *Journal of Business Research*, *69*(7), 2574-2584.

Cheung, R., & Jin, Q. (2016). Impact of a career exploration course on career decision making, adaptability, and relational support in Hong Kong. *Journal of Career Assessment*, *24*(3), 481-496.

Gure, G. S. (2015). Different scale construction approaches used to attitude measurement in social science research. *International Journal of Research in Economics and Social Sciences*, *5*(1), 26-44.

Lamm, K. W., Lamm, A. J., & Edgar, D. (2020). Scale development and validation: Methodology and recommendations. *Journal of International Agricultural and Extension Education*, *27*(2), 24-35.

Lemrová, S., Reiterová, E., Fatěnová, R., Lemr, K., & Tang, T. L. P. (2014). Money is power: Monetary intelligence—love of money and temptation of materialism among Czech University students. *Journal of Business Ethics*, *125*, 329-348.

Lu, L., & Jia, Q. (2022). Do college students with future work self-salience demonstrate higher levels of career adaptability? From a dual perspective of teachers and students. *Frontiers in psychology*, *13*, 1011447.

Spector, P. E. (1992). *Summated rating scale construction: An introduction* (Vol. 82). Sage.

Wadsworth, M. E., & Compas, B. E. (2002). Coping with family conflict and economic strain: The adolescent perspective. *Journal of research on adolescence*, *12*(2), 243-274.

A3: Thank you for your insightful feedback regarding the inclusion of control variables in our study.

The primary reason for not introducing the control variables in the Introduction was to maintain focus on the relationships among the key variables of interest in our study and to highlight our contributions and the issues we aim to address. Our intention was to provide a clear and concise overview of the core research questions and hypotheses. While the Introduction emphasized the main variables and their interactions, it was necessary to include control variables in the Methods section to ensure the robustness and validity of our findings. These control variables, such as gender, age, educational grade, and the number of weekly courses, are known to potentially influence the relationships we are studying. Therefore, controlling for these variables helps to isolate the effects of the primary variables and provides a more accurate understanding of the phenomena under investigation.

Our approach is consistent with established research practices and recommendations from prior studies (e.g., Chen et al., 2023; Cheung & Arnold, 2010; Lu et al.,2024). By including these control variables, we aim to account for potential confounding factors that could otherwise skew the results.

Reference:

Chen, H., Liu, F., & Wen, Y. (2023). The influence of college students’ core self-evaluation on job search outcomes: Chain mediating effect of career exploration and career adaptability. *Current Psychology*, *42*(18), 15696-15707.

Cheung, R., & Arnold, J. (2010). Antecedents of career exploration among Hong Kong Chinese university students: Testing contextual and developmental variables. *Journal of Vocational Behavior*, *76*(1), 25-36.

Lu, L., Gao, X., & Xiao, Y. (2024). Impact of college students’ perceived teacher support on career exploration: A Self-Determination Theory approach. *Innovations in Education and Teaching International*, *61*(3), 528-540.

Discussion
The researchers mentioned discussions but failed to present anything under them.
More concrete examples of how the findings could be implemented in practice would be beneficial. The section could include more discussion on the practical significance of the findings, not just statistical significance.

A: Thank you for your valuable feedback on our research. We note the lack of specifics in the discussion section you mentioned, and suggest adding more concrete examples of practical applications of research findings and discussion of practical significance.

We have revised the practical implication in accordance with your suggestions to better demonstrate the practical application value of the research findings and to provide practical recommendations for educators, policy makers and career coaches. Thank you again for your valuable feedback, and we are confident that these improvements will significantly increase the practical application and impact of the research. (It's highlighted in blue in the manuscript)

**Thanks to the reviewer and editor's suggestion, we have done our best to revise the manuscript carefully. We hope that we succeeded in addressing your concerns and that you will now consider the paper suitable for publication in *Plos One*.**
