# Supplementary material for: Children from the poor families seem to grow up earlier: An examination of how family economy stress links to career exploration
Source: PLoS One. 2024 Oct 29;19(10):e0310866. doi: 10.1371/journal.pone.0310866 (PMC11521272; doi:10.1371/journal.pone.0310866)
Supplement: Attachment — Submitted filename: Response to Reviewers second.docx [file pone.0310866.s004.docx]

**Response to Reviewers**

Thank you for your valuable feedback. We have modified the manuscript according to your opinions and answered the questions raised by reviewers point by point all with the blue context like here. It is appreciated to be recognized by reviewers.

**Reviewer #2:**

The Manuscript is well written.

However, there are several issues that need to urgently be addressed.

A: Thank you for your thorough review and constructive feedback on my manuscript. I appreciate your positive comments and the recommendations provided to enhance the clarity and quality of the paper. Here is my response to the suggested revisions:

1. The Analysis should be backed up with the generally accepted thresholds and the appropriate citations for those limits.

A: Thank you for your detailed review and valuable suggestions on our paper. In our analysis, we have adopted several generally accepted thresholds and standards in various fields. For example, in statistical significance testing, we used a significance level of 0.05, which is widely recognized in social science research. Additionally, for measuring internal consistency, we used Cronbach's alpha coefficient and set 0.7 as the acceptable minimum, based on the recommendations by Nunnally (1978). In our specific analyses (including CFA, measurement, descriptive statistics and hypotheses testing), we have clearly marked the thresholds and standards used, and provided detailed literature citations in the corresponding sections of the text (e.g., Bauer et al., 2006; Dawson, & Richter, 2006; Dawson, 2014).

Reference:

Bauer, D. J., Preacher, K. J., & Gil, K. M. (2006). Conceptualizing and testing random indirect effects and moderated mediation in multilevel models: new procedures and recommendations. *Psychological Methods*, *11*(2), 142-163.

Dawson, J. F., & Richter, A. W. (2006). Probing three-way interactions in moderated multiple regression: development and application of a slope difference test. *Journal of Applied Psychology*, *91*(4), 917-926.

Dawson, J. F. (2014). Moderation in management research: What, why, when, and how. *Journal of Business and Psychology*, *29*(1), 1-19.

Nunnally, J. C. (1978). An overview of psychological measurement. *Clinical diagnosis of mental disorders: A handbook*, 97-146.

2. The results should display what exactly happened with your hypotheses, I suggest a Table of a suggestive way to depict this information from the manuscript.

A: Thank you for your insightful suggestion regarding the inclusion of a table to explain the status of our hypotheses. We appreciate your recommendation and acknowledge the value it could add to the clarity of our manuscript. We have added a table in the results section to present the specific outcomes of each hypothesis test in a clear and intuitive manner (in Table 9).

Table 9 *Results of hypotheses.*

| Hypothesis Number | Hypothesis Description | Test Method | Conclusion |
| --- | --- | --- | --- |
|  |  |  |  |
| H1 | Family economic stress positively predicts vocational students’ career exploration. | Regression Analysis | Supported |
| H2 | *Love of money mediates the relationship between family economic stress and* *career exploration.* | Mediated Regression Analysis | Supported |
| H3 | *Teacher support positively moderates the positive relationship between love of money and career exploration.* | Moderated Regression Analysis | Supported |
| H4 | *Effective part-time behavior positively moderates the positive relationship between love of money and career exploration.* | Moderated Regression Analysis | Supported |
| H5 | *Both vocational student perceived teacher support and effective part- time behavior positively moderate the indirect effect of family economic stress on* *career exploration through love of money.* | Multiple Regression Analysis | Supported |

3. The results need and adequate discussion in the light of previous literature findings.

A: Thank you for your valuable feedback on our research.

We have revised the discussion in accordance with your suggestions to better demonstrate the application value of the research findings and to provide practical recommendations for educators, policy makers and career coaches. Thank you again for your valuable feedback, and we are confident that these improvements will significantly increase the application and impact of the research. (It's highlighted in blue in the discussion of manuscript)

4. In regard with the Methodological approach, it should include:

- participants and procedure;

- measures that you used;

- analysis strategy;

- results where you need to enclose descriptive statistics and hypotheses testing.

A: Our study methods encompass several key components: Participants and Procedure, Ethics Approval and Consent to Participate, Measures, and Data Analysis. Additionally, we present the results of necessary descriptive statistics and hypothesis testing within the manuscript.

5. Have you performed a pilot study? please motivate your answer.

A: The primary motivations for performing the pilot study were as follows:

The pilot study allowed us to evaluate the feasibility of our research design and procedures in practice. This helped identify any potential issues in the study protocol and enabled us to make necessary adjustments before the main study (e.g., Lu et al., 2024; Lu & Jia, 2022; Zhang et al., 2024).

We utilized G*Power software to conduct a power analysis for our study. To perform this power analysis, we assumed an effect size (e.g., f^2 = 0.05 for multiple regression), an alpha level of 0.05, and our study's minimum required sample size, as determined by the power analysis for detecting a small effect size at this alpha level with adequate power, was identified to be at least 262 participants. Our actual sample size across the three waves was 600, surpassing this threshold.

Reference:

Lu, L., Gao, X., & Xiao, Y. (2024). Impact of college students’ perceived teacher support on career exploration: A Self-Determination Theory approach. *Innovations in Education and Teaching International*, *61*(3), 528-540.

Lu, L., & Jia, Q. (2022). Do college students with future work self-salience demonstrate higher levels of career adaptability? From a dual perspective of teachers and students. *Frontiers in psychology*, *13*, 1011447.

Zhang, C., Lu, L., Xiao, Y., Zheng, Z., Jia, Q., Luo, Y., & Luo, J. (2024). From the three-dimensional perspective of college students’ school, family and themselves, exploring the mechanism of proactive personality on career exploration. *International Journal for Educational and Vocational Guidance*, 1-22.

6. It is not clear which instruments have you been using and who designed it? Why have you been using a 5 point Likert scale instead of a 7 point?

A: Our study design and methods were all consistent with ethics approval and consent to participate, and our variable measures were widely used in multiple studies, demonstrating their internal consistency and construct validity. Furthermore, our methodology aligns with previous research.

We chose to use the 5-point Likert scale instead of the 7-point scale for the following reasons:

1. Studies have shown that fewer options (such as the 5-point scale) can reduce the cognitive load of respondents and improve the response rate and response quality of questionnaires. Respondents are more likely to quickly and accurately choose the option that best matches their feelings, thus reducing the possibility of neutral or random selection (Revilla, Saris, & Krosnick, 2014).

2. We selected the 5-point Likert scale to facilitate comparison with previous studies (e.g., Cai et a., 2015; Guan et al., 2013). Many studies in related fields have employed a 5-point scale, allowing us to directly compare results and enhance the external validity and consistency of our findings.

3. The 5-point Likert scale simplifies data processing, enabling clearer interpretation and reporting of results. Additionally, research has shown that the outcomes of using a 5-point scale are generally consistent with those obtained using a 7-point scale, with the former being more intuitively interpreted (Dawes, 2008).

By incorporating these considerations, we ensured that our choice of the 5-point Likert scale was both methodologically sound and practically beneficial.

Reference:

Cai, Z., Guan, Y., Li, H., Shi, W., Guo, K., Liu, Y., ... & Hua, H. (2015). Self-esteem and proactive personality as predictors of future work self and career adaptability: An examination of mediating and moderating processes. *Journal of Vocational Behavior*, *86*, 86-94.

Dawes, J. (2008). Do data characteristics change according to the number of scale points used? An experiment using 5-point, 7-point and 10-point scales. *International Journal of Market Research*, *50*(1), 61-104.

Guan, Y., Deng, H., Sun, J., Wang, Y., Cai, Z., Ye, L., & Li, Y. (2013). Career adaptability, job search self-efficacy and outcomes: A three-wave investigation among Chinese university graduates. *Journal of Vocational Behavior*, *83*(3), 561-570.

Revilla, M. A., Saris, W. E., & Krosnick, J. A. (2014). Choosing the number of categories in agree–disagree scales. *Sociological Methods & Research*, *43*(1), 73-97.

**Reviewer #3:** all comments have been duly attended to, however, the justification on why the authors did not add an argument for the control variables in the background and the framework has not been attended to enough. the authors should look at this comment and clarify it.

A: Thank you for your detailed review and valuable suggestions on our paper. We have apologized for not sufficiently addressing the justification for not adding an argument for the control variables in the background and framework. Here is our further clarification and explanation on this matter:

First, our study primarily focuses on the relationships between specific key variables. Adding too many control variables could potentially divert the focus of the research and obscure the clarity of the main conclusions. We prioritized variables that are most directly related to the research objectives to ensure the study's specificity and effectiveness.

Second, according to previous studies, we found that gender, age and education will affect individual career exploration (Cai et al., 2015). At the same time, we found that there are differences in career exploration behavior between courses weekly and support of college students (Cheung & Jin, 2016; Denault et al., 2019). We also control possible control variables to ensure the rigor and science of the study. Control variables are also considered in Statistical Analysis, and the results of our analytical data are all under the control of control variables.

In the actual data collection process, some potential control variables may not be included due to data availability constraints. This is an unavoidable aspect of practical research. Therefore, we chose control variables with high data integrity and reliability to ensure the robustness of our analysis results.

Final, our selection of control variables is based on relevant theories and support from previous research (e.g., Cai et al., 2015; Cheung & Jin, 2016; Denault et al., 2019; Guan et al., 2013). We referred to numerous related studies and selected variables that have been theoretically and empirically proven to have significant impacts. This not only strengthens the theoretical foundation of our model but also ensures the scientific and credible nature of our research results.

In summary, our selection of control variables results from comprehensive considerations, including research objectives, data availability, statistical analysis needs, and theoretical basis. We believe that this approach helps ensure the accuracy and reliability of our research findings.

Reference:

Cai, Z., Guan, Y., Li, H., Shi, W., Guo, K., Liu, Y., ... & Hua, H. (2015). Self-esteem and proactive personality as predictors of future work self and career adaptability: An examination of mediating and moderating processes. *Journal of Vocational Behavior*, *86*, 86-94.

Cheung, R., & Jin, Q. (2016). Impact of a career exploration course on career decision making, adaptability, and relational support in Hong Kong. *Journal of Career Assessment*, 24(3), 481-496.

Denault, A. S., Ratelle, C. F., Duchesne, S., & Guay, F. (2019). Extracurricular activities and career indecision: A look at the mediating role of vocational exploration. *Journal of Vocational Behavior*, 110, 43-53.

Guan, Y., Deng, H., Sun, J., Wang, Y., Cai, Z., Ye, L., & Li, Y. (2013). Career adaptability, job search self-efficacy and outcomes: A three-wave investigation among Chinese university graduates. *Journal of Vocational Behavior*, *83*(3), 561-570.

**Thanks to the reviewer and editor's suggestion, we have done our best to revise the manuscript carefully. We hope that we succeeded in addressing your concerns and that you will now consider the paper suitable for publication in *Plos One*.**
